# Asymmetric Schottky Barrier in Rubrene Transistor via Monolayer Graphene Insertion toward Self-Powered Imaging

**DOI:** 10.3390/ma16237364

**Published:** 2023-11-27

**Authors:** Qing Liu, Xialian Zheng, Mengru Li, Qianqian Du, Chunhui Zhu, Wenjun Wang, Shuchao Qin

**Affiliations:** 1 School of Physical Science and Information Engineering, Liaocheng University, Liaocheng 252059, China; 2220110708@stu.lcu.edu.cn (Q.L.); 2210110210@stu.lcu.edu.cn (X.Z.); 2120280409@stu.lcu.edu.cn (M.L.); duqianqian@lcu.edu.cn (Q.D.); wjwang@lcu.edu.cn (W.W.); 2 Key Laboratory of Optical Communication Science and Technology of Shandong Province, Liaocheng 252059, China; 3 Hebei Key Laboratory of Photophysics Research and Application, Hebei Normal University, Shijiazhuang 050024, China; chunhuizhu@hebtu.edu.cn

**Keywords:** rubrene, graphene, transistor, imaging

## Abstract

Organic semiconductor materials featuring lightweight, and flexibility may play a significant role in various future applications, such as foldable displays, wearable devices, and artificial skin. For developing high-performance organic devices, organic crystals are highly desired, while a remaining fundamental issue is their contact problem. Here, we have grown a high-quality rubrene single crystal by utilizing a simple in-air sublimation technique. The contact characteristics (barrier height and contact resistance) are detail-studied by resist-free transfer electrodes (Au metal or graphene/Au). The Schottky barrier of the rubrene/graphene interface is lower and can be also modulated by gate bias, which is confirmed by spatial photocurrent mapping. Finally, we demonstrated the zero-bias photocurrent imaging application by constructing the asymmetrical device employing different electrode contacts. Our work would be of significance for studying the contact issue of organic crystals and wireless imaging.

## 1. Introduction

Organic materials have attracted extensive attention due to their intrinsic advantages including light weight, excellent flexibility, and improved carrier transport [1,2]. They have been used in various fields such as photovoltaic cells, light-emitting diodes, memories, and photon sensors [3,4,5,6]. By virtue of their incomparable light absorption (>10^5^ cm^−1^) and long photoexcited carrier lifetime, organic thin-film photon sensors have been intensively investigated in the last few years, showing their promising applications in sensitive and polarized photodetectors [7,8,9]. However, the inferior carrier mobility and limited exciton diffusion length of the thin film state hamper the photoelectrical conversion properties [10].

As an ideal candidate, organic crystals with a large domain size brought a leap in electrical mobility and exciton diffusion [10]. However, one of the remaining fundamental issues in organic devices has been the electrode contact issue. Numerous experiments have demonstrated that the contacts between organic materials and metal materials (such as Au, Ag, and Pt) are generally the Schottky contact regardless of the energy level (or work function) of metal materials [11,12]. Especially regarding channel length scaling, the metal–semiconductor (M–S) contact issue featured by the Schottky barrier becomes a crucial factor limiting the device scaling and performance. Therefore, the main charge transport tends to obey the Schottky–Mott law in organic transistors. A number of experimental results indicate that the barrier height is mainly influenced by this Fermi-level pinning and other external environmental factors such as dipole layers. In addition, the traditional metal deposition technique involves some chemical and structural disorders, and metal-induced gap states, resulting in Fermi-level pinning [13,14]. Compared with the typical metal deposition process, the nondestructive van der Waals (vdW) physically transferred electrodes have been demonstrated to achieve better contact in two-dimensional (2D) materials [15,16,17,18] and it is also more preferred in organic devices. For the organic pentacene film device, the enhanced charge injection efficiency has been reported by employing graphene electrodes in comparison to conventional Au electrodes. Graphene has revealed numerous unique physical and electrical properties [19,20], including high mobility and transparent flexibility, making it suitable as an electrode [21]. Its chemical potential and Fermi level can be controlled by the gate voltage [22]. Therefore, the physical properties of the device at the metal–semiconductor interface will be regulated by the gate voltage. Recently, Zeng et al. realized an ultralow contact for organic semiconductors by using transferred high-work function platinum electrodes through enhancing M–S orbital hybridization [13]. While it is a pity that the contact issue and photoelectrical performance are still scarce for organic crystals as compared to organic films or 2D material systems.

Rubrene is a star material for studying organic semiconductor-based devices, demonstrating high carrier mobility [23,24], a large absorption coefficient, and outstanding photoresponses [25]. The small-molecule nature enables their crystallization through various growth techniques, such as physical vapor transport (PVT), solution method, and microspacing in-air sublimation [26,27,28]. The in-air sublimation is an expedient growth technique with no need for the cost equipment, vacuum and/or carrier gas environment and complex procedure, so it provides a good platform to study the fundamental physical characteristics and contact interfaces for organic semiconductors.

In this paper, we fabricated the rubrene single crystal-based transistor device by using the microspacing in-air sublimation method, as well as the resist-free transfer electrode technique, and studied the contact interface between rubrene crystals and electrodes (Au and graphene). The Schottky barrier can be reduced by inserting a monolayer graphene at the rubrene crystal/Au metal interface, while the contact resistance shows an inverse augmentation trend. The spatial photocurrent mapping confirmed the barrier difference between rubrene/graphene and rubrene/Au, and also manifested that the rubrene/graphene Schottky barrier is gate bias-dependent. Finally, we demonstrated the zero-bias photocurrent imaging applications using this asymmetrical barrier configuration, for different wavelength-stimulated light.

## 2. Materials and Methods

### 2.1. Crystal and Device Fabrication

**Figure 1:**
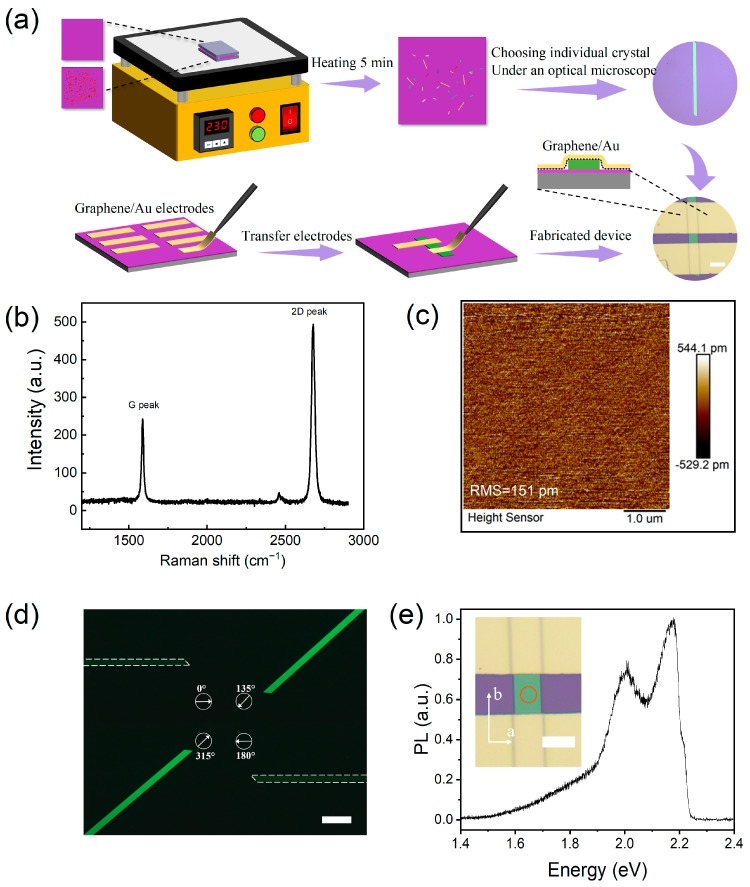
Characteristics of the material and device. (**a**) The detailed process of rubrene single crystal growth and device fabrication. Optical images of typical devices are shown in the flowchart. Scale bar: 40 µm. (**b**) Raman of the graphene. (**c**) The surface topography of rubrene single crystal. (**d**) Orthogonal polarized optical microscope images under different rotation angles. Scale bar: 40 µm. (**e**) Photoluminescence spectroscopy of the rubrene single crystal. The inset shows the PL test area on the optical microscope image and the orientation of the crystal’s a and b axes. Scale bar: 20 µm.

Rubrene powder was purchased from a commercial company (Alfa Aesar, Shanghai, China) without further purification. The growth method of the rubrene single crystal and the fabrication process of the device is illustrated in Figure 1a. Some rubrene powders (~3 mg) were put dispersedly on the bottom substrate. The top substrate was placed upside down, with the bottom substrate with a tiny space (~300 μm) between them. The rubrene single crystal was grown on the top SiO_2_/Si substrate by heating the bottom substrate to sublime rubrene materials, and the growth temperature and time were about 230 °C and 5 min. We adopted a ‘low-energy’ transfer electrode technology. Unlike ‘high-energy’ metal deposition processes, the resist-free transfer technique uses the van der Waals integration to form the organic material/electrode interface. This method eliminates the need for direct chemical reaction bonding, avoids related chemical disorders and the gap states caused by defects, and retains the inherent electronic properties of organic single crystals. Firstly, the chemical vapor deposition monolayer graphene is transferred on the SiO_2_/Si substrate. Then, the Au is prepared onto the monolayer via vacuum thermal evaporation to form the graphene/Au stripes, as shown in Figure 1a. As depicted in the flowchart of Figure 1a, the prefabricated monolayer graphene/Au stripes and pure Au electrode (reference experiment) were mechanically transferred onto the rubrene surface by using the tungsten micro-probe tip under the optical microscope, serving as the source/drain electrodes, respectively. The cross-sectional view of the device with transferred graphene/Au electrodes is also shown in Figure 1a. 

### 2.2. Materials and Device Characterizations 

The optical properties were characterized via Zeiss Imager A2m microscopy (Carl Zeiss, Oberkochen, Germany). The surface morphology was performed by Bruker Dimension Icon (Bruker, Hamburg, Germany) atomic force microscopy (AFM). Raman and PL measurements were performed using a 532 nm excitation laser, ×100 objective lens with about 1 µm diameter spot size. For the optical characteristics of the device, we used 405 and 532 nm laser diodes, respectively.

## 3. Results and Discussion

The Raman finger of graphene on the SiO_2_/Si substrate is shown in Figure 1b, indicating its defect-free monolayer nature. As shown in Figure 1c, we used the AFM technique to investigate the surface morphology of rubrene. The root-means-square (RMS) roughness is only 151 pm, indicating its considerable flatness. Conjugated π bonds in rubrene molecules enable its great light absorption ability and outstanding charge transport [29], making it an ideal choice for exploiting high-performance organic optoelectronic devices. For rubrene single crystals, the π–π overlap along the *b*-axis direction is the strongest, so the channel length of the device is parallel to the *b*-axis to obtain the best charge transport. The detailed molecular structure, arrangement as well as the direction of *a*- and *b*-axes can be seen in Figure S1 (Supporting Information). 

The anisotropy of rubrene single crystals allows us to study their optical properties using polarization optical microscopy (POM). Figure 1d shows the POM image of a typical rubrene crystal under a 532 nm LED light source [30]. Figure 1d shows the steady-state photoluminescence spectrum (PL) characterization from the ab face of the rubrene sample. There are two PL peaks at the range of 1.4–2.4 eV, where the stronger peak located at 2.15 eV, which originated from the luminescence emission of the (001) plane, which is exactly corresponding to the forbidden band gap. The other PL peak at 2.02 eV originated from the luminescence side emission of the (001) plane and reflects its strong optical anisotropy [31]. The weak high-energy shoulder at 2.22 eV implies the high surface quality of rubrene.

**Figure 2:**
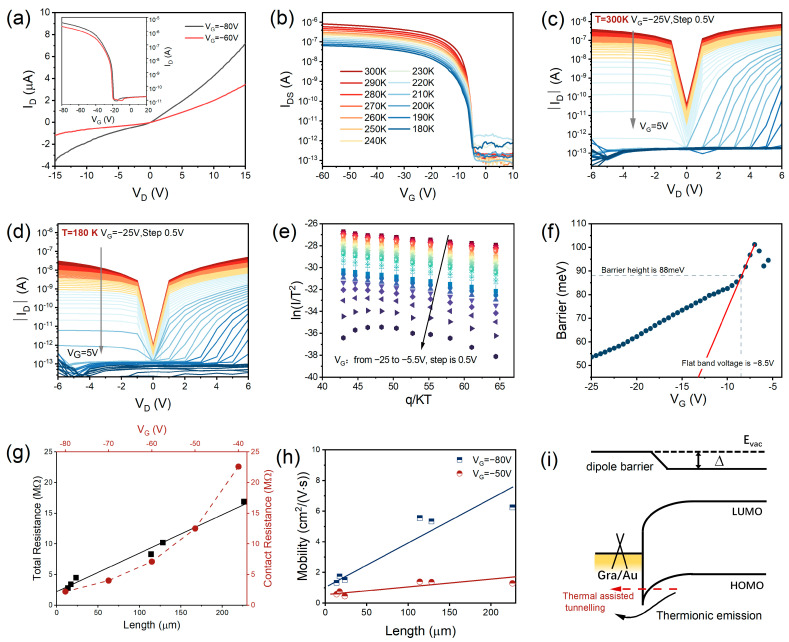
Electrical properties of the devices. (**a**) The output curve of the device with the graphene/Au electrode in the ambient environment. Inset shows the transfer curves of the device at V_D_ = −15 V. (**b**) Transfer curves (at V_D_ = −5 V) of the device under different temperatures in vacuum. (**c**) Output curves under different gate biases at room temperature (300 K). (**d**) Output curves under different gate bias at a low temperature (180 K). (**e**) Arrhenius plot of the device with graphene/Au electrodes, at V_D_ = −5 V. (**f**) Schottky barrier extracted from subfigure (**e**). The Schottky barrier height at the flat-band voltage is about 88 meV. (**g**) Total resistance (black square) for different channel lengths and contact resistances (brown circle) as the function of gate bias. (**h**) Mobility values extracted as the function of channel length. (**i**) Energy band diagram for charge transport.

**Figure 3:**
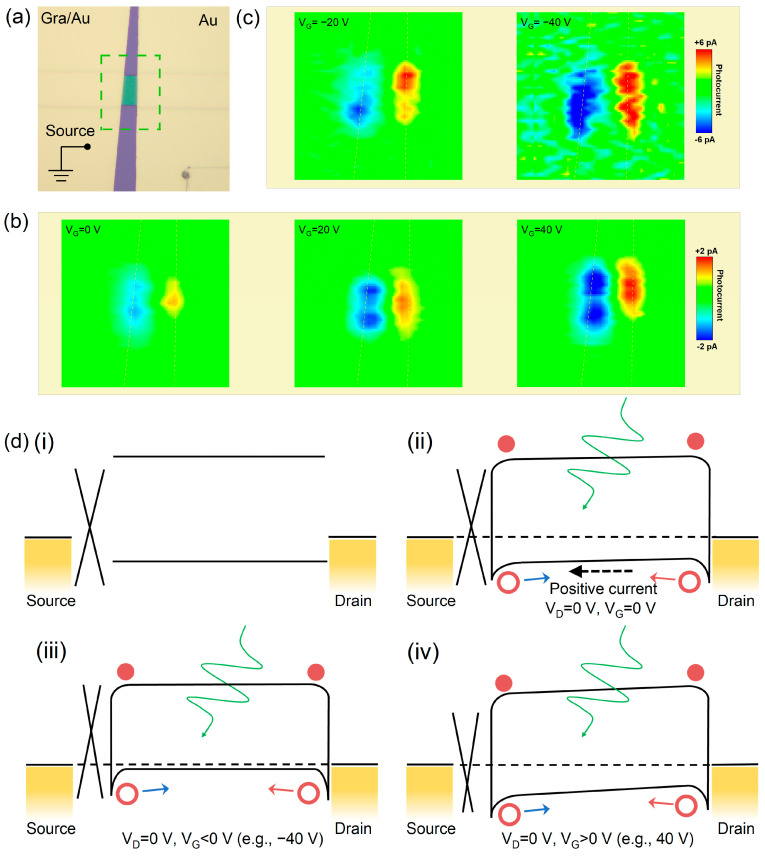
Photocurrent mapping and band diagrams. (**a**) Optical image of the device with asymmetrical electrodes. The scanning area is marked by a green box. (**b**) The photocurrent mapping at zero bias, under zero and finite positive gate bias, using a 532 nm laser. (**c**) The photocurrent mapping at zero bias, under finite negative gate bias. (**d**) Schematic band diagrams before contact (**i**) and after contact under zero gate bias (**ii**), negative gate bias (**iii**), and positive gate bias (**iv**). The red circles (bullets) represent hole (electron) respectively.

The typical output curves (I_D_–V_D_) of the device with graphene/Au electrodes are shown in Figure 2a, and the corresponding transfer curves are shown in the inset of Figure 2a. The asymmetrical I_D_–V_D_ curve is a typical feature of the Schottky contact between the channel material and electrodes, and it originated from the asymmetrical barrier height under the drain bias. Then, we quantitatively evaluated the contact characteristics using the variable temperature experiment. From the temperature-dependent transfer curves in Figure 3b, we can see that the drain current is strongly related to the temperature, showing a diminishing trend with a decrease in the temperature. Figure 2c shows the output curves of the device using graphene/Au electrodes under different gate biases at a high temperature (300 K). For the positive gate bias region, these I_D_–V_D_ curves show the obvious nonlinear and asymmetric character, implying the existence of the Schottky barrier. With the increase in gate bias to the negative region, it transferred into the symmetrical behavior due to enough carrier concentration and reduced Schottky barrier height. At low temperatures (e.g., 180 K as shown in Figure 2d), the output curves exhibited a more obvious distinct character, certifying the Schottky contact. To further explore the effect of gate voltage and temperature on the contact characteristics and carrier transport, we plotted the relation of ln(*I*/*T*^2^) versus *q*/*k_B_T* at a fixed drain bias of −5 V (Figure 2e). The data can be reasonably fitted by a linear plot, indicating that the primary charge transport of the device obeys thermal excitation theory: $J=-A^{*}T^{2}\exp\left(\frac{-q\Phi_{eff}}{k_{B}T}\right)$, where $A^{*}$ is the effective Richardson constant, *q* is the element charge, *T* is the Kelvin temperature, *k_B_* is Boltzmann constant, and Φ*_eff_* is the Schottky barrier height.

By fitting the slope of ln(*I*/*T*^2^) versus *q*/*k_B_T*, the barrier Φ*_eff_* can be extracted under different gate biases, as shown in Figure 2f. The barrier height increases linearly from the negative region (−25 V) to the positive region, while it turns a corner at the gate bias of -8.5 V which is called the flat-band voltage (V_FB_) [32]. When V_G_ is equal to V_FB_, the effective barrier height (Φ_B_) is about 88 meV. In our opinion, the total current of the device is dominated by the thermionic emission and thermally assisted tunneling current. When the V_G_ is lower than V_FB_ (|V_G_| < 8.5 V), the thermionic emission current is dominant because the thermally assisted tunneling current is limited by the high barrier [33]. The drain current should be rewritten as: $I_{D}=A^{*}T^{2}\exp(-\frac{q\Phi_{eff}-a\sqrt{V_{D}}}{k_{B}T})$, with $a=q\sqrt{\frac{q\Phi_{eff}}{4\pi \varepsilon_{0}\varepsilon d}}$, where *ε* is the dielectric constant of rubrene and *d* is the thickness of rubrene. With the increase in V_G_ to the negative region (|V_G_| > |V_FB_|), the thermally assisted tunneling current becomes non-negligible due to reduced Schottky barrier height and increasing carrier concentration. We also performed a reference experiment for a device with pristine Au electrodes using the same rubrene sample, and the detailed results are shown in Figure S2 (Supporting Information). With the decrease in temperature, the drain currents exhibited a similar downward trend. The barrier height corresponding to the flat band voltage was about 159 meV, which is higher than that of the device with graphene electrodes.

For a semiconductor device, the total resistance (*R_total_*) would be composed of metal electrode resistance (*R_m_*), contact resistance (*R_c_*), and channel resistance of the semiconductor material (*R_semi_*): $R_{total}=2R_{m}+2R_{c}+R_{semi}$. For our organic device, the channel resistance with several micron lengths was much larger than that of Au resistance, so the above formula can be simplified to: $R_{total}=2R_{C}+R_{sheet}\frac{L}{W}(R_{semi}=R_{sheet}\frac{L}{W})$, where the *R_sheet_* is the sheet resistance relying on the channel length. Therefore, the contact resistance can be extracted through the *R_total_* when the channel is close to zero, which is usually called the transfer length method (TLM) [34]. By linear scale fitting the resistance scatter with different channel lengths, the cross-value of the scatter line and *Y*-axis (experimental resistance) indicates the contact resistance in which the channel length is zero. Next, we assessed the contact resistance employing this method, and the total resistance as the function of the channel length is shown in Figure 2g. The corresponding optical microscope image of the under-test device produced by TLM can be seen in Figure S1 (Supporting Information). From Figure 2g, we can calculate that the contact resistance is about 2.2 MΩ under −80 V gate voltage. Meanwhile, the *R_c_* would change with the gate bias, and it shows a downward trend with the decrease in gate bias from −40 to −80 V. This phenomenon should be attributed to the higher carrier density, *n*, because *R_c_* is usually a direct proportion to $1/\sqrt{n}$. Mobility can be calculated using the following formula in the linear region: $\mu =\frac{\partial I_{D}}{\partial V_{G}}\frac{L}{W}\frac{1}{C_{ox}V_{D}}$, where *L* and *W* are the channel length and width, *C_ox_* is the oxide capacitance of 11.5 nF·cm^−2^, $\partial I_{D}/\partial V_{G}$ = *g_m_* is the transconductance, *V_D_* is the drain bias to the source terminal of −10 *V*, and *V_G_* is the gate voltage. For the long channel device, the effect of contact resistance is suppressed, so we calculated better carrier mobility in Figure 2h. Likewise, such consistency in electrical properties was also observed in the device with pristine Au electrodes; the test results are provided in the Supporting Information Figures S3 and S4. The contact resistance is lower than the device with the graphene/Au electrode. This comparison result is opposite to the performance of the Schottky barriers in the two devices.

This asymmetrical Schottky barrier is also certified by the spatial photocurrent mapping technique. We constructed an asymmetrical rubrene transistor where one contact is graphene/rubrene and another one is the Au/rubrene interface, as shown in Figure 3a. Firstly, we performed the photocurrent mapping at zero drain bias. For the zero driving field, the obvious photocurrents with opposite directions appeared near two electrodes, as shown in Figure 3b, confirming the existence of the photovoltaic effect due to the Schottky barrier (built-in field in semiconductors). The Fermi level of the graphene is more sensitive than that of the rubrene semiconductor, due to its finite density of states near the Dirac point [35]. As the gate voltage increases in the positive direction, the Fermi level of graphene rises, resulting in a larger built-in field. Therefore, we found an increased photocurrent near the graphene/rubrene interface under a higher positive gate bias. When the gate voltage is >40 V, the graphene Fermi level remains almost constant and independent of gate voltage, as shown in Figure S5. On the contrary, the decreased photocurrent should be theoretically obtained in the negative gate voltage region. But we reversely gained an increased photocurrent, which should be attributed to the decreasing contact resistance and increasing efficient hole density in the negative gate voltage region. Based on these experimental results, the photocurrent can be explained by a qualitative band diagram in Figure 3d.

**Figure 4:**
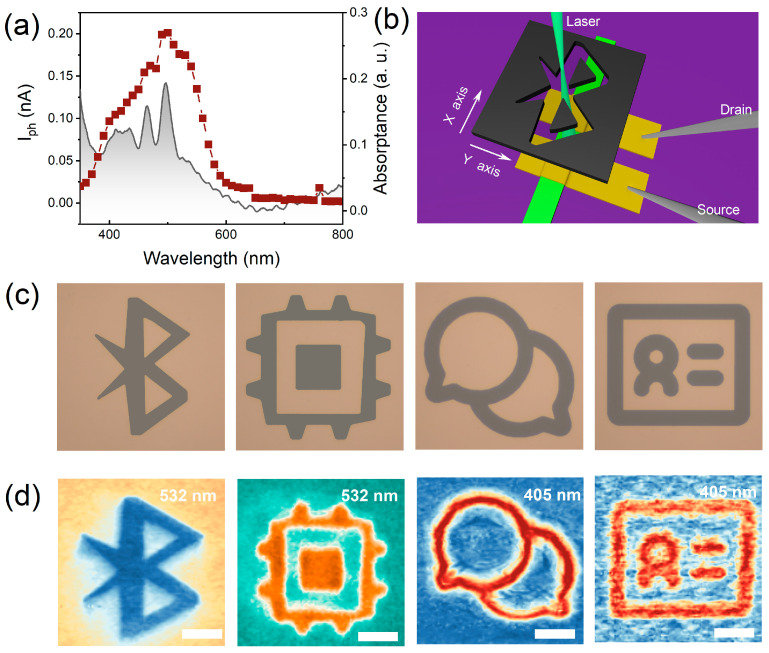
Broadband photoresponse and imaging. (**a**) The photocurrent of the asymmetrical device for different incident wavelengths (Red curve), and the bottom shaded area corresponds to the light absorption of the rubrene crystal on the quartz substrate. (**b**) Schematic diagram of the single-pixel imaging measurement system. (**c**) The patterned masks, including Bluetooth, chip, WeChat, and IC card. (**d**) Photocurrent imaging results under 532 and 405 nm lasers. Scale bar: 20 μm.

The above asymmetrical Schottky barrier allows us to execute self-powered sensor imaging, which plays an increasingly important role in today’s society, including environment monitoring, wireless sensing, and medical imaging. Firstly, we traced the broadband response of a typical asymmetrical rubrene transistor from 350 to 800 nm, as shown in Figure 4a. From here, we can see that the rubrene transistor exhibited good photoresponse from the whole visible light range. The responsivity peaks at the visible range (380–580 nm) are consistent with the interband absorption of the rubrene crystal, as shown in the shaded area in Figure 4a. To demonstrate the multiple imaging, we set up a single-pixel imaging system employing the lasers of 405 and 532 nm as exciting light, as illustrated in Figure 4b. The metal mask patterned target object is placed between the light source and the sensor device. The relative position between the light source and the device is fixed, and the metal mask can be moved linearly in the X–Y plane controlled by two piezo tubes. For a defined position, the current under dark and light illumination is recorded to obtain the position-dependent photocurrent. Therefore, we can remodel the pattern information of the target object by using the photocurrent. Figure 4c shows several optical images of the undertest patterned masks. Figure 4d shows the corresponding zero-bias high-resolution imaging information consisting of 100 × 100 scanned steps for 405 and 532 nm modulated laser signals, respectively. These results suggested that such asymmetrical configuration is promising as a self-powered imaging block building in the future wireless imaging system.

## 4. Conclusions

In conclusion, we fabricated rubrene single crystals using the microspacing in-air sublimation method. The Schottky barrier of the rubrene crystal/Au electrode can be improved by introducing monolayer graphene, while it is still Schottky type rather than the ohmic contact, and the charge transport is dominated by the thermionic emission and tunneling mode. The variation in the Schottky barrier can also be confirmed by the spatial photocurrent mapping. Finally, we demonstrate several self-powered imaging by using this asymmetrical barrier configuration. Our work provides some significant reference for studying organic crystals and zero-bias imaging.

## Data Availability

The data presented in this study are available on request from the corresponding author.

## Supplementary Materials

The following supporting information can be downloaded at: https://www.mdpi.com/article/10.3390/ma16237364/s1, Figure S1: Molecular structure and device; Figure S2: Arrhenius plot and Schottky barrier of the device with Au electrodes; Figure S3: Output curves and transfer curves of the device with Au electrodes; Figure S4: Contact resistances and mobility of the device with Au electrodes; Figure S5. The photocurrent mapping at zero bias, using a 532 nm laser.

## References

[1] Wang C., Dong H., Jiang L., Hu W. (2018). Organic Semiconductor Crystals. Chem. Soc. Rev..

[2] Chen Z., Obaid S.N., Lu L. (2019). Recent Advances in Organic Optoelectronic Devices for Biomedical Applications. Opt. Mater. Express.

[3] Tvingstedt K., Dal Zilio S., Inganaes O., Tormen M. (2008). Trapping Light with Micro Lenses in Thin Film Organic Photovoltaic Cells. Opt. Express.

[4] Wang R., Wang T., Kang Z., Zhang H., Yu R., Ji W. (2022). Efficient Flexible Quantum-Dot Light-Emitting Diodes with Unipolar Charge Injection. Opt. Express.

[5] Ren Z., Yang J., Qi D., Sonar P., Liu L., Lou Z., Shen G., Wei Z. (2021). Flexible Sensors Based on Organic-Inorganic Hybrid Materials. Adv. Mater. Technol..

[6] Wu L., Fukuda K., Yokota T., Someya T. (2019). A highly responsive organic image sensor based on a two-terminal organic photodetector with photomultiplication. Adv. Mater..

[7] Zhu D., Jiang W., Ma Z., Feng J., Zhan X., Lu C., Liu J., Hu Y., Wang D., Zhao S. (2022). Organic Donor-Acceptor Heterojunctions for High Performance Circularly Polarized Light Detection. Nat. Commun..

[8] Wu D., Guo J., Du J., Xia C., Zeng L., Tian Y., Shi Z., Tian Y., Li X.J., Tsang Y.H. (2019). Highly Polarization-Sensitive, Broadband, Self-Powered Photodetector Based on Graphene/PdSe_2_/Germanium Heterojunction. ACS Nano.

[9] Zhang C., Xu C., Chen C., Cheng J., Zhang H., Ni F., Wang X., Zou G., Qiu L. (2022). Optically Programmable Circularly Polarized Photodetector. ACS Nano.

[10] Dursun I., Guzelturk B. (2021). Exciton Diffusion Exceeding 1 μm: Run, Exciton, Run. Light Sci. Appl..

[11] Diao L., Daniel Frisbie C., Schroepfer D.D., Paul Ruden P. (2007). Electrical Characterization of Metal/Pentacene Contacts. J. Appl. Phys..

[12] Gundlach D.J., Zhou L., Nichols J.A., Jackson T.N., Necliudov P.V., Shur M.S. (2006). An Experimental Study of Contact Effects in Organic Thin Film Transistors. J. Appl. Phys..

[13] Zeng J., He D., Qiao J., Li Y., Sun L., Li W., Xie J., Gao S., Pan L., Wang P. (2023). Ultralow Contact Resistance in Organic Transistors via Orbital Hybridization. Nat. Commun..

[14] Schweicher G., Garbay G., Jouclas R., Vibert F., Devaux F., Geerts Y.H. (2020). Molecular Semiconductors for Logic Operations: Dead-End or Bright Future. Adv. Mater..

[15] Liu Y., Weiss N.O., Duan X., Cheng H.-C., Huang Y., Duan X. (2016). Van Der Waals Heterostructures and Devices. Nat. Rev. Mater..

[16] Liu L., Kong L., Li Q., He C., Ren L., Tao Q., Yang X., Lin J., Zhao B., Li Z. (2021). Transferred van Der Waals Metal Electrodes for Sub-1-Nm MoS2 Vertical Transistors. Nat. Electron..

[17] Liu Y., Huang Y., Duan X. (2019). Van Der Waals Integration before and beyond Two-Dimensional Materials. Nature.

[18] Wang X., Pan L., Yang J., Li B., Liu Y., Wei Z. (2021). Direct Synthesis and Enhanced Rectification of Alloy-to-Alloy 2D Type-II MoS_2(1-*x*)_Se_2*x*_/SnS_2(1-*y*)_Se_2*y*_ Heterostructures. Adv. Mater..

[19] Meng Q., Chen F., Xu Y., Cheng S., Yang W., Yao D., Yi Z. (2023). Tunable Terahertz Double Plasmon Induced-Transpare based on monolayer patterned graphene structure. Photonic Nanostruct..

[20] Silva B., Rodrigues J., Sompalle B., Liao C.-D., Nicoara N., Borme J., Cerqueira F., Claro M., Sadewasser S., Alpuim P. (2021). Efficient ReSe_2_ Photodetectors with CVD Single-Crystal Graphene Contacts. Nanomaterials.

[21] Wang X., Zhi L., Muellen K. (2008). Transparent, Conductive Graphene Electrodes for Dye-Sensitized Solar Cells. Nano Lett..

[22] Meng Q., Chen F., Xu Y., Cheng S., Yang W., Yao D., Yi Z. (2023). Multi-Frequency Polarization and Electro-Optical Modulator Based on Triple Plasmon- Induced Transparency in Monolayer Graphene Metamaterials. Diam. Relat. Mater..

[23] Chen Y., Lee B., Fu D., Podzorov V. (2011). The Origin of a 650 nm Photoluminescence Band in Rubrene. Adv. Mater..

[24] Du Q., Qin S., Wang Z., Gan Y., Zhang Y., Fan L., Liu Y., Li S., Dong R., Liu C. (2021). Highly Sensitive and Ultrafast Organic Phototransistor Based on Rubrene Single Crystals. ACS Appl. Mater. Interfaces.

[25] Pei K., Wang F., Han W., Yang S., Liu K., Liu K., Li H., Zhai T. (2020). Suppression of Persistent Photoconductivity of Rubrene Crystals using Gate-Tunable Rubrene/Bi_2_Se_3_ Diodes with Photoinduced Negative Differential Resistance. Small.

[26] Ye X., Liu Y., Han Q., Ge C., Cui S., Zhang L., Zheng X., Liu G., Liu J., Liu D. (2018). Microspacing In-Air Sublimation Growth of Organic Crystals. Chem. Mater..

[27] Gan Y., Qin S., Du Q., Zhang Y., Zhao J., Li M., Wang A., Liu Y., Li S., Dong R. (2022). Ultrafast and Sensitive Self-Powered Photodetector Based on Graphene/Pentacene Single Crystal Heterostructure with Weak Light Detection Capacity. Adv. Sci..

[28] Ye X., Liu Y., Guo Q., Han Q., Ge C., Cui S., Zhang L., Tao X. (2019). 1D Versus 2D Cocrystals Growth via Microspacing In-Air Sublimation. Nat. Commun..

[29] Wang C., Dong H., Hu W., Liu Y., Zhu D. (2012). Semiconducting π-Conjugated Systems in Field-Effect Transistors: A Material Odyssey of Organic Electronics. Chem. Rev..

[30] Zhao J., Zhu Q., Li X., Liu Y., Li S., Wang W., Wang F., Qin S. (2023). Self-Powered Organic Phototransistors with Asymmetrical van Der Waals Stacking for Flexible Image Sensors. ACS Photonics.

[31] Irkhin P., Ryasnyanskiy A., Koehler M., Biaggio I. (2012). Absorption and Photoluminescence Spectroscopy of Rubrene Single Crystals. Phys. Rev. B.

[32] Lee S., Tang A., Aloni S., Wong H.-S.P. (2016). Statistical Study on the Schottky Barrier Reduction of Tunneling Contacts to CVD Synthesized MoS_2_. Nano Lett..

[33] Chen J., Calvet L.C., Reed M.A., Carr D.W., Grubisha D.S., Bennett D.W. (1999). Electronic Transport through Metal-1,4-Phenylene Diisocyanide–Metal Junctions. Chem. Phys. Lett..

[34] Kim Y., Chung S., Cho K., Harkin D., Hwang W.-T., Yoo D., Kim J.-K., Lee W., Song Y., Ahn H. (2019). Enhanced Charge Injection Properties of Organic Field-Effect Transistor by Molecular Implantation Doping. Adv. Mater..

[35] Castro Neto A.H., Guinea F., Peres N.M.R., Novoselov K.S., Geim A.K. (2009). The Electronic Properties of Graphene. Rev. Mod. Phys..

