# Supplementary material for: Asymmetric Schottky Barrier in Rubrene Transistor via Monolayer Graphene Insertion toward Self-Powered Imaging
Source: Materials (Basel). 2023 Nov 27;16(23):7364. doi: 10.3390/ma16237364 (PMC10707313; doi:10.3390/ma16237364)
Supplement: Supplementary file 1 [file materials-16-07364-s001.zip › materials-2673662-supplementary.pdf]

Asymmetric Schottky Barrier in Rubrene Transistor via Monolayer Graphene Insertion toward Self-Powered Imaging

Qing Liu [1,2], Xialian Zheng [1,2], Mengru Li [1,2], Qianqian Du [1,2], Chunhui Zhu [3], Wenjun Wang [1,2] and Shuchao Qin [1,2,*]

[1] School of Physical Science and Information Engineering, Liaocheng University, Liaocheng 252059, China
[2] Key Laboratory of Optical Communication Science and Technology of Shandong Province, Liaocheng 252059, China
[3] Hebei Key Laboratory of Photophysics Research and Application, Hebei Normal University, Shijiazhuang 050024, China
* Correspondence: qinshuchao@lcu.edu.cn

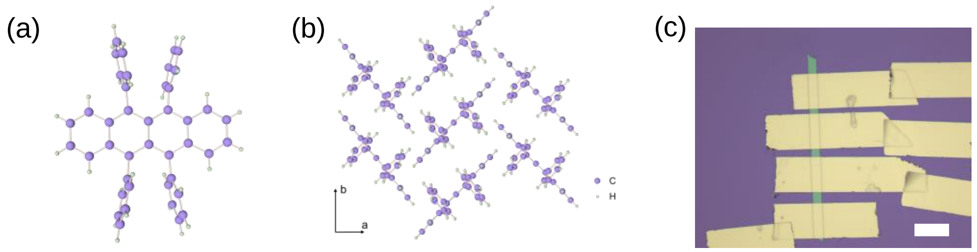

Figure S1. (a) Molecular structure of rubrene single crystal. (b) Molecular arrangement of rubrene crystal. (c) The optical microscope image of the device. Four electrodes are transferred on the rubrene crystal for the TLM calculation. Scale bar: 80 μm.

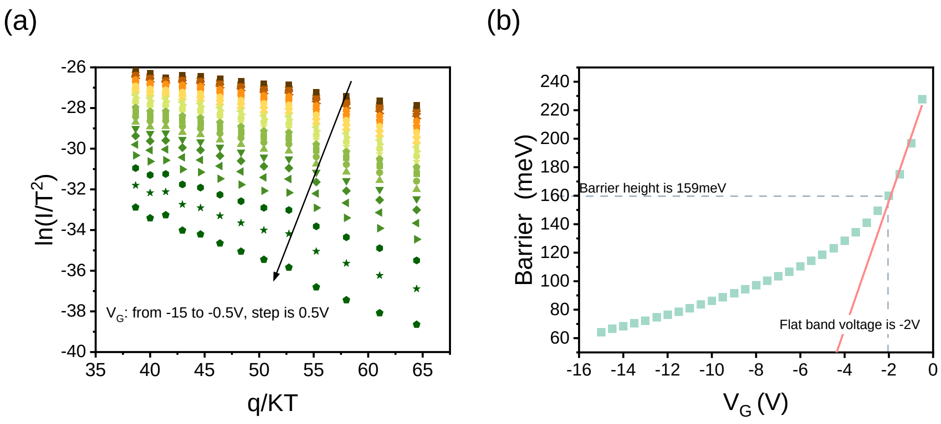

Figure S2. (a) Arrhenius plot of device with Au electrodes, at V_D = -5 V. (b) Schottky barrier extracted from the figure **a**.

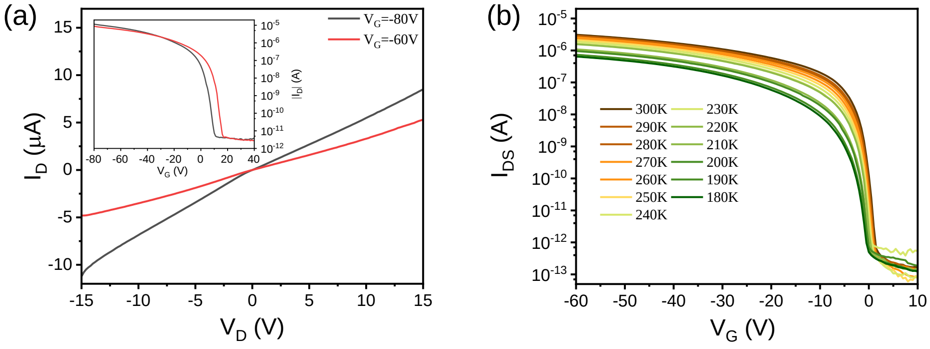

Figure S3. (a) The output curve of device with Au electrodes at ambient environment. Inset shows transfer curves of device at V_D = -15 V. (b) Transfer curves of the device under different temperatures in vacuum environment, at V_D = -5 V.

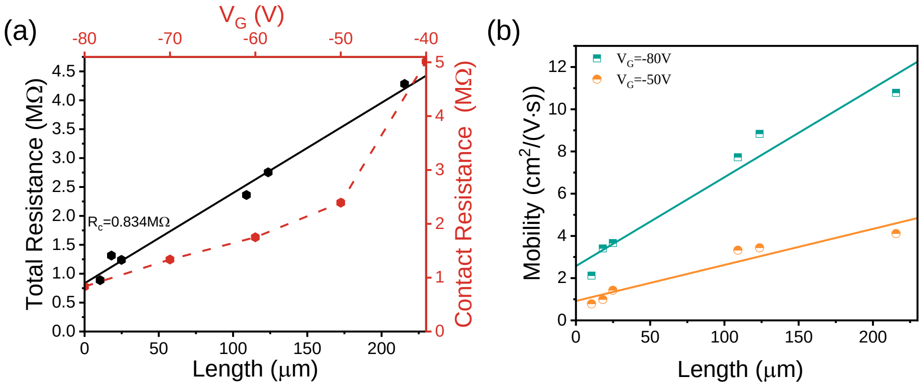

Figure S4. (a) Total resistance (black hexagon) for different channel length, and the contact resistances (red hexagon) as the function of gate bias. (b) Mobility values of the device with Au electrodes as the function of channel length.

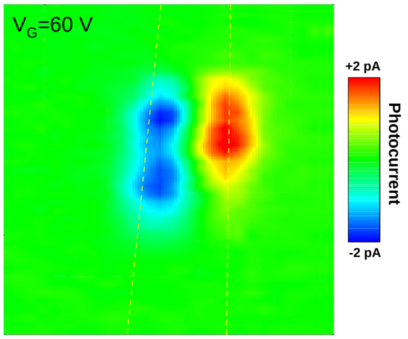

Figure S5. The photocurrent mapping at zero bias, using a 532 nm laser.
